# Demographic and Socioeconomic Factors Associated to Fruits and Vegetables Consumption in Elderly Europeans: A Systematic Review

**DOI:** 10.3390/ijerph20043442

**Published:** 2023-02-15

**Authors:** Malak Kouiti, Carmen Ortega-Rico, Juan Pedro Arrebola, Mabel Gracia-Arnaiz, Cristina Larrea-Killinger

**Affiliations:** 1 Department of Preventive Medicine and Public Health, Universidad de Granada, 18071 Granada, Spain; 2 Laboratory of Health Sciences and Technologies, Higher Institute of Health Sciences, Hassan First University of Settat, Settat 26000, Morocco; 3 Instituto de Investigación Biosanitaria (ibs.GRANADA), Avda. de Madrid, 15. Pabellón de Consultas Externas 2, 2a Planta, 18012 Granada, Spain; 4 Consortium for Biomedical Research in Epidemiology and Public Health (CIBERESP), Instituto de Salud Carlos III, C/Monforte de Lemos 3-5, Pabellón 11. Planta 0, 28029 Madrid, Spain; 5 Department of Anthropology, Philosophy and Social Work, Universitat Rovira i Virgili, Av., 43002 Tarragona, Spain; 6 Department of Social and Cultural Anthropology, University of Barcelona, 08001 Barcelona, Spain; 7 Food Observatory (ODELA), University of Barcelona, 08001 Barcelona, Spain

**Keywords:** elderly people, fruits and vegetables consumption, socioeconomic factors, demographic factors, systematic review

## Abstract

Several epidemiological studies stress the association between a diet based on high fruits and vegetables intake and a better health condition. However, elderly Europeans cannot manage the recommended fruits and vegetables consumption. This systematic review aims to explore the main factors related to fruits and vegetables consumption in elderly Europeans. We conducted literature searches on Medline, Scopus, and Web of Science from inception to May 2022. Published articles including data related to certain fruits and vegetables consumption among elderly Europeans were selected. The New Castle-Ottawa Scale and National Heart, Lung, and Blood Institute tools were used for methodological quality assessment by two authors independently. A total of 60 articles were retrieved, and data from twenty-one high-quality cross-sectional studies and five moderate-to-high-quality cohort studies, including a total of 109,516 participants, were synthesized. Associated factors mostly analyzed were those relating to demographic and socioeconomic status, such as sex, age, marital status, educational level, and income. However, the findings show a high discrepancy. Some evidence suggests a possible positive association, while other evidence shows an inverse or no association at all. The relationship between demographic and socioeconomic factors with fruits and vegetables consumption is not at all clear. More epidemiological studies with an appropriate design and corresponding statistical methods are required.

## 1. Introduction

In the next 20 years, an increase by about 45% of the population aged 65 years and over is expected in Europe [1]. This demographic change will have an effect on the incidence of chronic diseases, considering the association between age and multiple health outcomes such as cardiovascular, endocrine, and neurological diseases and osteoporosis [2,3]. Therefore, this situation calls for a major effort to ensure quality of life in our older population. Covering basic needs such as ensuring the consumption of healthy food is the basis for dignified and independent ageing. 

Several epidemiological studies stress the importance of decreasing the consumption of simple carbohydrates and increasing high-fiber intake, as well as maintaining frequent physical activity, to prevent chronic diseases and improve human health [4,5]. Dietary patterns with high fruits and vegetables consumption, such as the Mediterranean diet, are associated with a decreased incidence of cardiovascular and endocrine diseases [6,7]. In this sense, a high daily intake of fruits and vegetables in a varied dietary pattern seems to prevent multiple chronic conditions such as cardiovascular disease, kidney disease, metabolic diseases such as obesity and type 2 diabetes, as well as depression [8,9,10,11]. 

At present, the World Health Organization (WHO) recommends a daily intake of five portions of fruits and vegetables [12]. However, only 12% of the European population comply with the five fruits and vegetables consumption per day, and 26% of people over 65 years do not eat any servings of fruits and vegetables. Women are the ones who better comply with the recommendations [13]. 

Multiple factors can be related to a greater or lesser consumption of fruits and vegetables. Some studies stress a possible relationship with sex, income, or age, as various health problems such as tooth loss, dysphagia, and cognitive problems develop over the years [14]. However, this association differs in other studies. Considering that diet can be critical for modulating the risk of chronic diseases, this systematic review aims to identify the main associated factors with low fruits and vegetables consumption among elderly Europeans.

## 2. Materials and Methods

This review is reported according to the 2020 update of Preferred Reporting Items for Systematic Reviews and Meta-analysis (PRISMA) (Supplementary material; Table S2). 

### 2.1. Eligibility Criteria and Research Strategy 

This review was conducted to explore the main factors associated with fruits and vegetables consumption. The research strategy was built according to the PECOS statement:

- **P**opulation: non-institutionalized elderly people aged between 55 and 80 years old;
- **E**xposure: factors associated to fruits and vegetables intake (gender, age, SES, etc.);
- **C**omparators: lowest fruits and vegetables intake;
- **O**utcome: fruits and vegetables consumption (quantity and variety);
- **S**tudy design: quantitative and qualitative observational studies.

Three databases, Medline, Scopus, and Web of Science, were used for the research. The selection of the included studies was conducted based on previously set selection criteria: (1) quantitative and qualitative observational studies including cross-sectional and cohort design; (2) published from the inception of each database to May 2022; (3) in Spanish, English, or French; (4) conducted in Europe; (5) including non-institutionalized elderly population without specific diseases; (6) analyzing factors associated to fruits and vegetables consumption. Gray literature, books, communications, and reviews were excluded. 

The research equation was established using the following keywords:

- Elderly, older, aged, aging, senior*;
- Fruits intake, vegetables intake, fruits consumption, vegetables consumption, food intake, food consumption, healthy food, healthy eating, healthy diet, food deserts;
- Income, lower class, social class, economic, vulnerability*, social, resource, socioeconomic, poverty, inequality*;
- Household, living standard, living alone, cohabiting, loneliness, residence, social relations;
- Occupational level, occupation, employment, retirement*;
- Educational level, education;
- Gender, sex, woman*, man*;
- Ethnic, race.

### 2.2. Quality Assessment

The quality of the selected studies was assessed independently by two authors (M.K. and C.O.-R), and discrepancies were solved through discussion with a third researcher. In accordance with the study design, three assessment tools were employed. Cross-sectional studies were evaluated using National Heart, Lung, and Blood Institute (NIHLBI) tools [15]. Two items related to a longitudinal design were not considered for cross-sectional studies: (1) sufficient time frame to see an association and (2) participants who did not engage in follow-up. Twelve items related to the research question, selection of participants, participation rate, exposure, and outcome assessment, and confounders control was also assessed. Classification of the studies was carried out based on the following cut-off scores: “0–4, low quality”, “5–9, moderate quality”, and “10–12, high quality”. For cohort studies, the Newcastle–Ottawa tool was used to assess the level of quality [16]. The scale consists in assessing 8 items related to selection, comparability, and outcome. A maximum of 9 stars can be assigned to each study (2 stars can be assigned to the item of comparability). The considered scores were “≥8 stars, high quality”, “6 to 7, moderate quality”, “≤5 stars, low quality”.

### 2.3. Data Extraction and Data Synthesis 

Two reviewers (M.K. and C.O.-R) performed the data extraction using a predefined form. The principal information included country, year of publication, study design, sampling strategy, sample size, eligibility criteria, participant characteristics, principal outcome (dietary pattern, diet quality, fruits and vegetables quantity), assessment methods, exposure (associated factors such as socioeconomic factors, educational level, age, sex, and others), controlling factors, and principal outcomes.

Different factors associated with higher or lower consumption of fruits and vegetables were explored, and a narrative synthesis of the consulted articles was conducted for high-quality cross-sectional studies and moderate-to-high-quality cohorts. 

## 3. Results

### 3.1. Literature Research

**Figure 1:**
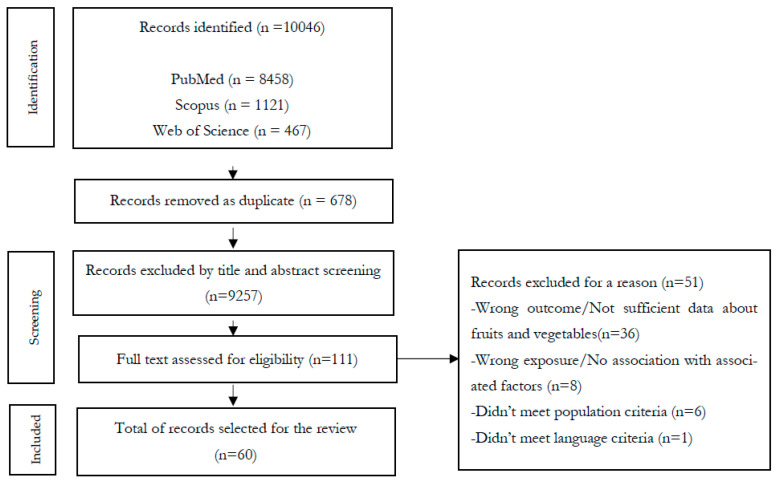
Flow chart: process applied to select the included studies.

A total of 10,046 articles were retrieved. After removing duplicates, title and abstract screening was carried out, and 111 articles meeting the eligibility criteria were selected for full-text screening (further details in Table S1. Supplementary Material). Finally, 60 records published between 1995 and 2021 were included in our review (Figure 1). 

### 3.2. Quality Assessment of Included Studies

**Figure 2:**
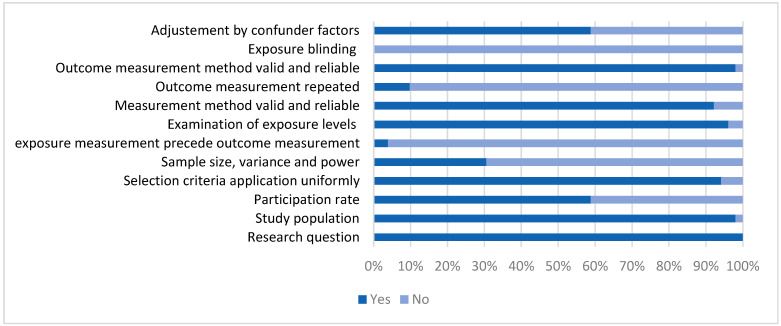
Completion of cross-sectional studies using NHLBI quality assessment tool.

Methodological quality was assessed for 60 selected records. The NHLBI quality tool was applied for 51 cross-sectional studies, and the Newcastle–Ottawa Scale for nine cohorts. Regarding cross-sectional studies, 41.21% (*n* = 21) showed high quality, and 58.8% (*n* = 30) moderate quality. Weaknesses were mostly observed for the following items: (1) sample-size justification, power, variance, and effect estimation; (2) exposure measurement before the outcome measurement; (3) measurement of the outcome more than once, (4) blinding exposure (Figure 2 and Table S3). From the nine cohort studies assessed, only one showed high quality, while the percentage of low and moderate quality was similar at 44.44% (*n* = 4) (Table 1).

### 3.3. Characteristics of the Studies

For data synthesis, cross-sectional studies of high quality were analyzed. Regarding cohort studies, moderate-to-high-quality articles were examined as only one study showed high quality. A total of 26 articles were analyzed, including five cohorts and twenty-one cross-sectional. The mean characteristics of cohort studies were summarized in Table 2, and those of cross-sectional ones in Table 3. The distribution of studies according to the geographic area was as follows: five studies were conducted in the United Kingdom (UK) [18,19,26,27,28], five in the Netherlands [29,30,31,32,33], four in France [20,22,34,35], two in Spain [36,37], Greece [38,39] and Portugal [40,41], and only one study was conducted in each of these countries: Finland, Switzerland, Germany, Italy, and Norway. Finally, one study was multisite, conducted in France, Italy, and the UK. 

Outcomes were averaged by fruits and vegetables consumption or adherence to fruits and vegetables guidelines. Demographic factors were the principal exposure analyzed in eighteen articles. Eleven records report an association between socioeconomic status (SES) and fruits and vegetables consumption. Sample size ranged from 3392 [19] to [22] in cohort studies, and from 98 [37] to [29] in cross–sectional studies.

### 3.4. Association between Demographic Determinants and Fruits and Vegetables Consumption

The association between age and fruits and vegetables consumption is not at all clear. Some studies show an inverse association [20,29,36], other studies report no association at all [35,44]. The effect of age on fruits and vegetables is not the same [45]. Moreover, outcomes can differ depending on geographic area and sex [41,43]. Regarding sex, being female was associated with high fruits and vegetables consumption [20,26,31,33,37]. However, two records do not report any association [39,43]. The findings from one study suggest a significant high fruits consumption in men [45]. Regarding marital status and social isolation determinants, we were not able to clearly define the relationship with fruits and vegetables consumption. High variation was observed in the social situation definition, which makes the comparison less evident. Regarding geographic determinants, fruits and vegetables consumption can be significantly different when comparing countries or regions [28,43]. However, the differences between rural and urban areas are not clear (Table 4). 

Other determinants were collected for this inventory (psychological state, smoking habits, cooking skills, and chewing ability). The available data were not sufficient to establish a conclusion regarding these factors (Table S4). Dijkstra et al. stress the price of fruits and vegetables and taste preferences as the principal barriers related to consumption [32]. Wandle’s findings relate fruits consumption to preferences, and vegetables consumption to nonfeasting meal patterns [47]. 

### 3.5. Association between Socioeconomic Status (SES) Predictors and Fruits and Vegetables Consumption

Outcomes related to the association between SES predictors and fruits and vegetables consumption were inconsistent. Some studies show a positive association between educational level [23,24,29,38], economic situation [24,37,39], and fruits and vegetables consumption. However, other studies show an inverse association between educational level and fruits and vegetables consumption [22,41] and no association with economic status [30,45]. Similarly, discrepancies were observed for employment status [17,22]. Outcomes can differ depending on sex [41,44] or geographic area [43] (Table 5).

## 4. Discussion

This review proposes an inventory of 21 high-quality cross-sectional studies and five cohort studies with moderate to high quality reporting about associated factors to fruits and vegetables consumption in elderly people. The main associated factors analyzed in the available scientific evidence were age, sex, and determinants related to marital status and SES. Although the association between these determinants and fruit and vegetable consumption may be clear in some age groups, such as in children and adolescents, this association shows greater diversity in older Europeans. On the other hand, other factors such as liking, accessibility, psychological changes, functional disabilities and health consciousness, and knowledge and awareness of current recommendations were suggested as possible factors [49,50]. 

A scoping review suggests some differences in fruits and vegetables consumption especially by geographic area, socioeconomic status, marital status, and gender. Fruit and vegetables consumption varies widely according to geographical area. In some countries such as the United States, Thailand, and Baltic countries, living in a rural area was negatively associated with fruits and vegetables consumption [50,51]. However, this association does not seem accurate in all European populations. Several countries, especially those in the Mediterranean basin, such as Italy, Spain, and Greece, show high fruits and vegetables intake [43,49]. Being married and a high socioeconomic status were positively associated with fruits and vegetables consumption. Regarding gender, women tend to comply better with the recommendations than men do [14]. On the other hand, although gender shows a difference in consumption among the younger group, this relationship was not distinctive among the oldest group [51]. Articles excluded for quality show similar results regarding variability in the association of determinants such as gender and geographic area on fruits and vegetables consumption. A comparison between geographic areas, defined as countries or regions, shows some differences [52,53]. However, no difference was observed between rural and urban areas [54]. 

Our conclusions related to advanced age were similar to those reported previously for adults [55]. However, outcomes from other studies show a potential positive association between determinants such as income, educational level, and physical activity [27,56,57,58]. Nevertheless, no difference was observed for age, gender, and smoking status in a cross-sectional study of 504 Iranian older adults [58]. These outcomes are opposed to Baker et al.’s findings, as their outcomes on 1024 UK older adults confirm that men consume less fruits and vegetables than women do [59]. 

This inconsistency between various findings may be explained by the particular characteristics of participants, as belonging to different groups according to geographic area, income level, education, and culture. Determinants are typically affected by other factors. Cooking skills in British men was associated to a better consumption of fruits and vegetables [60], although other factors such as living alone may influence the ability to cook. Moreover, this cannot be generalized to other cultures or countries. On the other hand, living in some geographic areas showing less fruits and vegetables intake, such as a rural area or shanty town, is usually related to the economic situation and educational level. Differences between countries may also be related to cultural factors, as would be the case with the Mediterranean diet.

Other factors may be related to the applied study design and statistical method. Furthermore, this relationship does not look the same for fruits as it does for vegetables. This fact further complicates the comparison, since some studies evaluate fruits and vegetables together and others, separately. Finally, like Niklett et al., we also observed a deficiency in studies that specifically analyze the factors associated to fruits and vegetables consumption [14]. 

Our systematic review has some strengths: (1) the research was extensive and included a large list of possible associated factors, (2) only high-quality evidence was included in the results, (3) reporting review was conducted according to the PRISMA checklist. However, some limitations must be taken into account when interpreting the results. At the methodological level, the review protocol was not published previously, and the included articles were not selected in pairs. However, quality assessment and data extraction were performed by two authors independently. The fact that the associated factors were self-reported by participants is another limitation to take into account. Furthermore, it should be noted that different patterns of diet can be observed depending on the stage of aging: young-old or old-old [61].

Identifying the consumption patterns and associated factors allows us to determine population groups at risk of nutritional deficiencies. This would help to guide public health campaigns in promoting healthy lifestyles and habits. Our findings provide a summary of data obtained in the high-quality research. However, conducting second-level epidemiological studies is a necessity in order to draw more definite conclusions regarding the factors associated to fruits and vegetables consumption. Longitudinal studies may help to define a causal pathway. 

## 5. Conclusions

Our review provides a summary of moderate-to-high-quality scientific evidence reporting data about potential associated factors to fruits and vegetables intake in elderly Europeans. Fruits and vegetables consumption may be associated to the analyzed predictors. However, confirmation of the presence of a causal relationship was not feasible due to the high level of inconsistency in the available findings. To draw stronger conclusions, more studies with an adequate design and hypothetical constructs such as health consciousness, psychological changes, independence level, and availability and diversity in local shops would be required to explore the factors associated to fruits and vegetables consumption. 

## Figures and Tables

**Table 1:** Newcastle–Ottawa Scale assessment of the cohort studies analyzing factors associated to fruits and vegetables consumption.

	Selection	Comparability	Outcome	Quality
Ali-Kovero et al., 2020 [17]	****	**	**	High
Boehm et al., 2018 [18]	***	**	**	Moderate
Kobayashi el al., 2018 [19]	***	**	**	Moderate
Plessz et al., 2017 [20]	***	**	**	Moderate
Vinther et al., 2016 [21]	**	**	*	Low
Plessz et al., 2015 [22]	***	**	**	Moderate
Svenja et al., 2005 [23]	***	None	*	Low
Del Pozo et al., 2003 [24]	****	None	*	Low
Schroll et al., 1997 [25]	**	**	*	Low

The asterisks Corresponds to stars described in the methodology of newcastle Ottawa scale.

**Table 2:** Characteristics of cohort studies analyzing the factors associated to fruits and vegetables consumption.

1st Author, Year (Country)	Sample Size	Eligibility Criteria	Participant Characteristics	Dietary Pattern/Comparators	Pattern Analysis Method	Associated Factors Analyzed	Controlling Factors	Outcome
Ali–Kovero et al., 2020 [17] (Finland)	6887	Employees who had turned 40, 45, 50, 55, or 60	- Age: women: retired 59.8 (2.6), employed 49.8 (5.5). Men: retired 59.7 (2.9), employed 50.3 (5.7) - Household income (euros/month): women: retired 2866 (1328), employed 3007 (1331). Men: retired 3307 (1278), employed 3244 (1265)	fruits, vegetables, and fish consumption	FFQ	Employment status	Age, marital status, limiting long-standing illness, and household income	- Vegetables consumption 1: women: retired 33.2 (15.8), employed 33.5 (16.3). Men: retired 27.0 (14.7), employed 26.3 (14.7) - Fruits consumption 1: women: retired 33.8 (17.0), employed 30.3 (17.4). Men: retired 22.4 (15.6), employed 19.8 (14.8) - Fish consumption 1: women: retired 7.7 (5.9), employed 7.2 (5.9). Men: retired 7.3 (5.6), employed 6.9 (5.6)
Boehm et al., 2018 [18] (England)	6565	Population aged 50 years and older	Age average: 65.0 (9.78); 55.4% women; 98.2% white	Fruits and vegetables intake	Interview and self-completion questionnaire	Psychological well-being (low, moderate, high)	Age, BMI, gender, race, socioeconomic status, education, marital status, diseases, smoking, and physical activity	NA
Kobayashi et al., 2018 [19] (England)	3392	Population aged 50 years and older	Age groups: 39% (52–59) and 38% (60–69); 44% are males; 65% overweight/obese; 71% married or living as married; 15% have a high degree of loneliness	Five daily fruits and vegetables intake.	Interview and self-completion questionnaire. Loneliness: UCLA	Social isolation and loneliness	Age, sex, sociodemographic factors, health indicators, social isolation and loneliness mutually, depression and depressive syndrome	24% complete with 5 daily fruits and vegetables intake. 25% and 17% from low social isolation and high social isolation groups, respectively. 25% and 21% with low loneliness and high loneliness, respectively
Plessz et al., 2017 [20] (France)	14,019	40–49 years in 1989 who reported living with a partner at that time and who were still in the cohort in 2014	Over 80% of complete-case respondents (92% of women respondents) had initially worked in subordinate positions (men were usually manual workers; women, office workers). The women had lower educational attainment (18% were secondary school graduates, as compared to 47% of the men). In 2014, 88% of respondents were living with a partner; only 3% had experienced more than two union dissolutions	Vegetables consumption	FFQ	Union dissolution (married vs. single)	Geographic area, age, whether retired, presence of children in the home, and 1990 questionnaire	55.1% of women ate vegetables every day in 2014 vs. 31.2% in 1990. 36.2% of men ate vegetables every day in 2014 vs. 18.6% in 1990.
Plessz et al., 2015 [22] (France)	20,652	40–49 years, retired after the first FFQ in 1990 and before the last FFQ in 2009; (d) aged between 50 and 61 years at retirement. Never had long-standing illness or disabilities, according to company records	Mean age men and women: 5.9 (2.8) Medium educational level men/women: 53.4%/51.9% Low educational level men/women: 18.95%/31.9%	Eating vegetables every day	FFQ	Age in years and retirement status	Sex, prescribed diet, changes in spousal status, educational level	NA

UCLA: University of California Los Angeles Loneliness Scale.

**Table 3:** Characteristics of cross-sectional studies analyzing factors associated to fruits and vegetables consumption.

1st Author, year (country)	Sample Size	Eligibility Criteria	Participant Characteristics	Dietary Pattern/Comparators	Pattern a\Analysis Method	Associated Factors Analyzed	Controlling Factors	Outcome
López-González et al., 2021 [36] (Spain)	6647	Men aged between 55 and 75 years and women 60–75 years, with a body mass index between 27 and 40 kg/m^2^, who meet at least three MetS criteria: abdominal obesity for European individuals (WC of 88 cm in women and 102 in men), high triglyceride levels (150 g/dL; or drug treatment for elevated triglycerides), low HDL- cholesterol (<50 mg/dL in women and <40 mg/dL in men, or drug treatment for reduced HDL-c), high blood pressure (SBP 130 mm Hg or DBP 85 mm Hg, or antihypertensive drug treatment), or high plasma fasting glucose (100 mg/dL, or drug treatment for elevated glucose)	T1 (lowest) (*n* = 2523) Age: 64.54 ± 5.00 Women (39.95%) BMI, kg/m^2^: 32.63 ± 3.40 -Abdominal obesity, *n*(%): 2372 (94.02) -Educational level, *n* (%): primary school 1135 (44.99), secondary school 809 (32.07), tertiary school 579 (22.95) -Marital status, *n* (%): married 1913 (76.09), widowed 241 (9.59), divorced/separated 210 (8.35), others 150 (5.97), living alone 320 (12.71) -Employment status, *n* (%): retired 1414 (56.38), employed 583 (23.25), housekeeper 283 (11.28), others 228 (9.09) T3 (highest) (*n* = 1851) Age: 65.31 ± 4.66 Women: 59.27% BMI, kg/m^2^: 32.51 ± 3.51 Abdominal obesity, *n*(%): 1760 (95.08) -Educational level, *n*(%): primary school 981 (53.00), secondary school 475 (25.66), tertiary school 395 (21.34). - Marital status, *n*(%): married 1430 (77.38), widowed 206 (11.15), divorced/separated 140 (7.58), other 72 (3.90), living alone 219 (11.83) - Employment status, *n*(%): retired 1021 (55.34), employed 327 (17.72), housekeeper 348 (18.86), other 149 (8.08)	Fruits and vegetables variety and quantity, diet quality	FFQ	Lifestyle (smoking habit, physical activity, WHO exercise recommendations, sedentary behavior, sleeping)	Sex and age	Fruits and vegetables consumption, lowest vs. highest tertiles: 286.87 ± 3.83 vs. 439.41 ± 4.48 and 237.70 ± 2.28 vs. 383.87 ± 2.67, respectively
Apostolaki et., 2020 [38] (Greece)	463	Elderly people from Protection Centers able to understand and respond to the questionnaire independently	85% males and 15% females. Male mean age was 75.4 (6.1) years, and women mean age 74.4 (6.2). 40.5% of women and 19.1% of men were obese. 70% report primary education	Adherence to the Mediterranean diet, dietary intake	Med Diet Score, water balance questionnaire (WBQ)	Gender	Age, education, marital status, annual income, car ownership, growing vegetables and fruits (Med Diet Score analysis)	Mean of Med Diet Score: 31.9 (3.6). Energy intake was 1678 (412) kcal/day
Der Toorn et al., 2020 [29] (Netherlands)	12,985	Middle-aged and elderly participants of the Rotterdam cohort	Sex: 58%women vs. 42% men Median (IQR) age: 66 years (59–74) - Education: (68.9%) lower/intermediate - BMI median: 26.5 kg/m^2^ (IQR: 24.3–29.1)	Diet quality	FFQ	Seasonal variation	Sex, age, cohort, energy intake, physical activity, body mass index, comorbidities, and education	- Diet quality score: Winter median: 7 (6–8), Spring median: 7 (5–8), Summer: 7 (5–8), Autumn: 7 (6–8) - Overall median energy intake (Kcal/day): 1996 (1653–2392)
Rodrigues et al., 2018 [42] (Portugal)	2393	Non-institutionalized adults (65+ years old) living in private residences in the Portuguese Mainland and Islands (Madeira and Azores)	55.8% were female 65+ years Mean BMI of all older adults was 27.3 ± 5.2 kg/m^2^ 77.9% with household income below 1000 euros per month Lower levels of education were found among the oldest seniors	Dietary habits	FFQ	Age, geographic area	NA	83.8% consume fruits every day, 59.9% consume vegetables every day Lower consumption of fruits and vegetable sin the Azores (69.0%) and Madeira (73.9%) regions
Schoufour et al., 2018 [30] (Netherlands)	5434	All residents aged 55+ in the Ommoord district	3210 were female (59%) Age categories: males/females - 55–64 y: 43%/42% - 65–74 y: 40%/37% - 75–84 y: 15%/19% - ≥85 y: 1%/2% Smoking: males/females - Current smoker: 29%/19% - Nonsmoker: 71%/81% BMI: - normal weight: 40%/36% - overweight: 52%/44% - obesity: 7%/19% Household income: - <28,000: 25%/49% - 28,000–39,999: 34%/26% - 40,000–54,999: 28%/18% - >54,999: 13%/7% Education - Primary education: 24% males, 42% females - Lower secondary: 24%/32% - Intermediate: 37%/22% - Higher: 15%/4%	Diet quality	Dutch Healthy Diet Index	Socioeconomic indicators (income, education, occupation)	Sex, age, smoking status, BMI, physical activity level, total energy intake, and mutually adjusted for the other socioeconomic indicators	Fruits consumption was stable in 93% of participants throughout follow-up Vegetables consumption increased in 14% of participants and decreased in 12%
Appleton et al., 2017 [43] (France, Italy, UK)	497	Aged 65 years or older, able to come to the institution undertaking the research, and able to fully understand and complete the consent and questionnaire	Mean age (SD): 72.1 (6.7). Highest education level: - No qualifications: 198 (40%) - University degree: 47 (9%) Current/most recent employment: - Unemployed: 61 (12%) - Manual worker: 62 (13%) - Non-manual worker: 237 (48%) - Professional/management: 137 (28%)	Quantity and variety of vegetables consumption	Questionnaire self-reported.	Demographic predictors (gender, age, country of residence, highest educational qualification)	NA	Mean quantity of vegetables consumed was low (mean = 2.1–2.7 portions/day)
Jimenéz Redondo et al., 2016 [37] (Spain)	98	All non-institutionalized residents aged 80 years and above	- Mean age: 86.6 (5.0), 61.5% widowed, 19.4% had a secondary school education, 16.3% living alone	Food-group consumption and nutritional status (MNA)	Mini Nutritional Assessment (MNA)	Gender	NA	Consumption (g/day) men vs. women -Vegetables: 271.7 ± 211.3 vs. 220.3 ± 163.3 - Fruits: 337.2 ± 215.6 vs. 290.7 ± 218.1
Dijkstra et al., 2015 [32] (Netherlands)	1057	Age <80 years, independently living, cognitively well-functioning and still alive on January 1, 2007	The total sample included 1057 participants, 555 women and 502 men, with a mean age of 68.9 years (SD: 6.2) (Table 1). More than half of the respondents had both a middle level of education and a middle level of income	Fruits, vegetables, and fish guidelines	FFQ	Perceived barriers on fruits consumption	Sex, age, alcohol consumption, partner status, and the other SES indicators.	Main barriers perceived are related to price and preferences.
Dijkstra et al., 2014 [31] (Netherlands)	1057	Age <80 years, independently living, cognitively well-functioning and still alive on January 1, 2007	The sample included 555 women and 502 men, with a mean age of 68.9 years (SD: 6.2). More than half of the respondents had a middle level of education and a middle level of income, 32.3% had a middle level of occupational prestige	Fruits, vegetables and fish intake (adherence to guidelines)	Short FFQ	Socioeconomic status (SES) indicators (education, household income, and occupational prestige)	Sex, age, SES indicators, partner status, and alcohol consumption	Fruits consumption ≥2 pieces/day: 82.5%. Vegetables (≥200 g a day): 65.1%.
Oliveira et al., 2014 [41] (Portugal)	2485	Non-institutionalized inhabitants of Porto aged ≥18 years. Those aged ≥65 years with no cognitive impairment	Compared to women, men were significantly more educated, often blue-collar workers (married (80.9 versus 60.8%), current smokers (34.7 versus 17.7%), and regular physical exercise practitioners (41.1 versus 31.5%)	Inadequate fruits and vegetables consumption	FFQ	Sociodemographic, Lifestyle, and anthropometric predictors	Age, education, marital status, smoking status, regular physical exercise, and total energy intake	The proportion of consumers of <5 servings per day of fruits and vegetables was 49.2 versus 42.2% in women and men, respectively. In both sexes, the daily mean (SD) consumption of vegetables was significantly higher than that of fruits
Danon-Hersch et al., 2013 [44] (Switzerland)	1260	65–70 years old non-institutionalized population living in Lausanne	Age: 65–75 years Prevalence of overweight (BMI 25.0–29.9 kg/m^2^), obesity (BMI ≥30.0 kg/m^2^), and abdominal obesity was 53%, 24%, and 45% in men; 35%, 23%, and 45% in women	Eating habits	MNA	Age, living arrangements, financial difficulties, symptoms of depression and education level	Living alone, financial difficulties, symptoms of depression, education level and current smoking	Fruits or vegetables ≥twice/day: men 80.5% and women 90.1
Giuli et al., 2012 [45] (Italy)	306	65+ years old healthy, non- institutionalized and did not require day care and/or nursing support volunteers. Free of medication such as steroids, diuretics, anticonvulsants, anti-depressant drugs, antibiotics, antimetabolites, non-steroid anti-inflammatory drugs and micronutrient supplementation	The mean age of the sample was 76.91 (8.5) years. The level of education in male vs. females was 12.8% vs. 6.4% for middle/high education; 56.1% of females were widowed, while 76.7% of males were married 75.7% of men were overweight and obese vs. 44.2% of women. Resources were not sufficient for daily food shopping	Dietary habits and nutritional aspects	FFQ	Sociodemographic and anthropometric factors	No	Fruits are the most consumed food in 7.46 ± 2.6, raw vegetables in 7.02 ± 2.8, other cooked vegetables in 5.58 ± 1.8
Perna et al., 2012 [46] (Germany)	3942	Residents in the study region of Augsburg, Germany	Age: 61.8% ≤75 years Sex: 52.5% women Educational level: 68.3% low Household income: 64.0% low Resilience (%) mid and low vs. upper third: 67.2% vs. 32.8%. Physical activity: 86.9% high to moderate 28.4% living alone	Health behavior including fruits and vegetables consumption	Short version of the Resilience Scale (RS), phone interview	Resilience	Educational level, household income	- Low fruits and vegetables consumption: 91.3%. - High fruits and vegetables consumption: 8.7%
Katsarou et al., 2010 [39] (Greece)	1129	Age: 65+ years Non-institutionalized, non-CVD or cancer	Age: 74 years Sex: 47% male Living in urban areas: 62% Living alone: 28% Smoking current: 14% Physical activity: 36% BMI: 28.6 kg/m^2^	Adherence to the traditional Mediterranean diet	Mediterranean Diet Score	Socioeconomic status (SES)	Age, sex, place of residence, cohabiting, BMI, physical activity, smoking, presence of diabetes, HTA, and hypercholesterolaemia	Fruits consumption in the lowest vs. highest tertiles of SES: 20 ± 6.4 vs. 20 ± 6.7. Vegetables consumption in the lowest vs. highest tertiles of SES: 53 ± 36 vs. 58 ± 35
Holmes et al., 2008 [26] (UK)	234	65+ years Either living alone or with other(s) of retirement age	All men Household type: - Living alone: 59% - Living with other(s): 41% Main food shopping: - Large supermarket: 74% - Small supermarket: 26% Transport to main shop: - By car: 57% - Other: 43% Area of residence: - Suburban/rural: 81% - Urban: 19% Income percentage spent on food: - <30%: 77% - 30%: 23% Cooking skills of MFP (main food provider): - Better developed: 75% - Less developed: 25%	Foods consumed and nutrient intake	Dietary data collection used the 24 h recall “multiple pass” method repeated on nonconsecutive days	Social, physical, and other factors such as cooking skills, ability to chew, and isolation.	No	Men with better developed cooking skills as main food providers consume more vegetables (117 g vs. 76 g/day). Approximate consumption of vegetables and fruits among men with difficulties to chew vs. without difficulties: 80 g vs. 119 g/day and 62 g vs. 98 g/day, respectively
Samieri et al., 2008 [35] (France)	1724	Living in one of these three French cities (Dijon, Bordeaux, and Montpellier), aged 65+, and not institutionalized	Age mean (SD): men vs. women: 76.0 (4.97) vs. 76.8 (5.10) BMI mean (SD) men vs. women: 26.9 (3.59) vs. 26.1 (4.56) Primary or secondary education (%) men vs. women: 53.1% vs. 66.6 Married (%) men vs. women: 78.9% vs. 41.4	Dietary pattern	FFQ, a 24 h dietary recall	NA	NA	- Mean (SD) fruits serving per week, men vs. women: 13.1 (6.90) vs. 13.7 (7.03) - Mean (SD) cooked vegetables serving per week, men vs. women: 10.1 (4.72) vs. 10.2 (4.24) - Mean (SD) raw vegetables and salad serving per week, men vs. women: 9.3 (4.96) vs. 8.8 (5.46)
Elia et al., 2005 [28] (England)	1155	65+ years old Subjects were living freely within the community or in residential accommodation	NA	Malnutrition, nutrient status and nutrient intake	4 d period by using the weighed food intake method	Geographical factors	Age, gender, and domicile	NA
Larrieu et al., 2004 [34] (France)	9250	Subjects aged 65+ recruited in three French cities: Bordeaux, Dijon, and Montpellier	60.7% women Age (%): - 65–74 y: 5118 (55.3) - 75–84 y: 3597 (38.9) - ≥85 71 y: 535 (5.8) Educational level (%): - low: 2424 (26.2) - middle: 5115 (55.3) - high 1711: (18.5) Lifestyle (%): - alone: 3313 (35.8) - other: 5937 (64.2)	Dietary pattern (raw and cooked vegetables, fruits, and other foods)	Standardized brief FFQ	Sociodemographic factors (sex, age, educational level, lifestyle)	NA	Frequency consumption ≥once a day: - Raw fruits: 78.1% - Raw vegetables: 50.4% - Cooked fruits and vegetables: 68.7%
Rossum et al., 2000 [33] (Netherlands)	5406	Aged 55+. Living between 1990 and 1993 in a district of Rotterdam, The Netherlands	Mean (SD) age women vs. men: 68 (7) and 67 (8) years, respectively. 37% of the population had only attended primary school Men had a higher level of education than women did: 37.1% vs. 21.7%	Nutrient intake (micronutrients, macro, daily intake of some food such as fruits and vegetables, intake of beverages)	semiquantitative FFQ	Educational level and socioeconomic status	Age and gender	Fruits daily intake mean (SD) in grams/day, men vs. women: 206 (131) vs. 243 (131). Vegetables daily intake mean (SD) in grams/day, men vs. women: 221 (92) vs. 219 (123)
Johnson et al., 1998 [27] (UK)	445	Urban area: non-institutionalized individuals aged 65+ living within the Nottingham area. Rural area: non-institutionalized individuals aged 55+ living within the Nottinghamshire, Lincolnshire, and Leicester- shire areas	Sex (urban/rural) (%): Female: 53.8/58.9 Male: 46.2/41.1 Age (urban/rural) (%): 65–74: 62.8/63.3 75+: 37.2/36.7 Social class (urban/rural) (%): Professional: 2.5/7.3 Managerial: 16.5/39.7 Skilled non-manual: 22.7/23.6 Skilled manual: 37.7/17.4 Semiskilled: 15.1/6.0 Unskilled: 5.5/6	Fruits and vegetables consumption	FFQ	Socioeconomic, physical, and psychological factors (rural vs. urban; smoking, social engagement, sex, age, health score, social class)	Age, income, educational level, and social grade.	Comply with recommended five fruits and vegetables consumption/day, urban vs. rural area: 37% vs. 51%. Low fruits and vegetables consumption was particularly associated with being male, smoking, and having low levels of social engagement
Wandel et al., 1995 [47] (Norway)	1091	>15 years in Norway (only data related to participants ≥60 was included in our review)	NA	Dietary intake of fruits and vegetables	Personal interview and mail questionnaire	Factors limiting the consumption of fruits, vegetables, and potatoes	NA	52% of participants ≥60 years old comply with daily fruits consumption, and 40% with vegetables consumption

CVD: cardiovascular diseases; FFQ: Food Frequency Questionnaire; NA: not available.

**Table 4:** Association between demographic determinants and fruits and vegetables consumption.

1st Author, Year (Country)	Age	Sex	Marital Status/ Social Isolation	Geographic Position
López-González et al., 2021 [36] (Spain)	Significant difference	Females had higher variety in fruits and vegetables intake	Significant differences	
Apostolaki et., 2020 [38]		Females had higher daily intake of fruits and vegetables		
Kobayashi et al., 2018 [19] (England)			Social isolation was associated with less consumption of fruits and vegetables: RR, 0.80 (0.62–1.04). Loneliness was not associated with 5 fruits and vegetables intake: RR, 0.39 (0.75–1.15)	
Appleton K et al., 2017 [43] (France, Italy, UK)	Positive association in UK and French participants, no association for Italian participants	No association with vegetables consumption		Significant difference between countries and quantity of fruits and vegetables consumption. No effect on variety
Plessz et al., 2017 [20] (France)			Union dissolution reduced men’s vegetables consumption. Women’s consumption was more affected if the partner had relatively low socio-occupational status. Living with children was associated with lower vegetables consumption	
Jimenéz Redondo et al., 2016 [37] (Spain)		Men showed high intake of fruits and vegetables (not statistically significant)		
Plessz et al., 2015 [22] (France)	Age was positively associated with high daily vegetables consumption		Single status was associated with high vegetables consumption	
Oliveira et al., 2014 [48] (Portugal)	Age showed an inverse association with fruits and vegetables intake in women		Marital status showed direct association with fruits and vegetables consumption only in men	
Danon-Hersch et al., 2013 [44] (Switzerland)	No association	Male complied less than women did with fruits or vegetables (FV) ≥ twice/day	Living alone was associated with fruits or vegetables consumption ≥ twice/day in men	
Giuli et al., 2012 [45] (Italy)	“Raw vegetabless” and “Cooked vegetabless” consumption decreased with age. No change for fruits	Men showed a significantly higher consumption of fruits than women did		
Perna et al., 2012 [46](Germany)			No association between living status and fruits and vegetables consumption	
Katsarou et al., 2010 [39] (Greece)	No association	No association	No association	No association (rural vs. urban area)
Samieri et al., 2008 [35] (France)	No association			
Holmes et al., 2008 [26] UK			Eating alone at the table was associated with highest fruits and vegetables consumption	
Elia et al., 2005 [28] (England)				Lower fruits and vegetables intake in the northern region of England as compared to central and southern regions
Larrieu S et al., 2004 [34](France)	Age was associated with lower consumption of raw vegetables	Men consumed more raw vegetables, while women had higher consumption of raw fruits and cooked fruits or vegetables	Living alone was associated with low consumption of fruits and vegetables	
Rossum et al., 2000 [33] (Netherlands)		Women consumed more fruits than men did		
Johnson et al., 1998 [27] (UK)	Vegetables consumption decreased significantly with age	Low fruits and vegetables consumption was particularly associated with being male	Singleness was associated with lower fruits and vegetables consumption in men	Living in a rural area was associated with higher compliance with 5 fruits and vegetables consumption/day

**Table 5:** Association between socioeconomic status (SES) determinants and fruits and vegetables consumption.

1st Author, Year (Country)	Educational Level	Income/Affluence Score	Employment Status/Retirement
López-González et al., 2021 [36] (Spain)	Significant difference	No difference	No difference
Ali–Kovero et al., 2020 [17] (Finland)			Statutory retirement was associated positively with fruits consumption in men but negatively with vegetables consumption in women
Schoufour et al., 2018 [30] (Netherlands)	Positive association with vegetables consumption. None with fruits consumption	No association	
Appleton K et al., 2017 [43] (France, Italy, UK)	Positive association in French and Italian participants. No association in UK participants	Positive association between quantity and affluence score	No association
Plessz et al., 2017 [20] (France)			Employment position at age 35 was not associated with vegetables consumption
Plessz et al., 2015 [22] (France)	Inverse association with vegetables consumption		Retirement was significantly associated with vegetables consumption for men but not for women
Oliveira et al., 2014 [41] (Portugal)	Inverse association in women. No association observed in men		
Danon-Hersch et al., 2013 [44] (Switzerland)	Educational level was associated with fruits or vegetables consumption ≥ twice/day in men	Financial difficulties were associated with less fruits or vegetables consumption in men and women	
Giuli et al., 2012 [45] (Italy)	Educational level was significantly and positively correlated with fruits consumption	No association	
Katsarou et al., 2010 [39] (Greece)	People in the highest SES group consumed larger quantities of vegetables compared to those in the lowest SES group		
Larrieu S et al., 2004 [34] (France)	Educational level was positively associated with fruits and vegetables consumption		

## Data Availability

All data generated or analyzed during this study are included in the manuscript and its Supporting Information file.

## Supplementary Materials

The following supporting information can be downloaded at https://www.mdpi.com/article/10.3390/ijerph20043442/s1, Table S1 Search strategy, Table S2 Full text screening: exclusion reason, Table S3 Completary list of articles included for the NHLBI quality assessment, Table S4 Other factors associated to fruit and PRISMA 2020 Checklist.
