# Supplementary material for: Demographic and Socioeconomic Factors Associated to Fruits and Vegetables Consumption in Elderly Europeans: A Systematic Review
Source: Int J Environ Res Public Health. 2023 Feb 15;20(4):3442. doi: 10.3390/ijerph20043442 (PMC9962118; doi:10.3390/ijerph20043442)
Supplement: Supplementary file 1 [file ijerph-20-03442-s001.zip › ijerph-2099665-supplementary.pdf]

Supplementary material

Table S1. Search strategy

Search	Query	Pubmed	Web of Science	Scopus
#1	Elderly OR older OR aging OR senior	6 460 689	5 445 420	2 438 372
#2	Vegetable consumption" OR "vegetable intake" OR "fruit consumption" OR "fruit intake" OR "food intake" OR "food consumption	73 715	4 096	5829
#3	Social OR socioeconomic OR economic OR income OR gender OR sex OR household OR education* OR retire* OR loneliness OR occupation*	5 103 785	6 140 157	8 901 060
#1 AND #2 AND #3		8 458	1221	467

Table S2. Full text screening: exclusion reason

Exclusión reason	Authors	Titles
Wrong outcome or not sufficient data about fruit and vegetables	Rueter et al., 2020.	How Older Citizens in Germany Perceive and Handle Their Food Environment—A Qualitative Exploratory Study
	Azzolina et al., 2020	Nutrients and Caloric Intake Associated with Fruits, Vegetables, and Legumes in the Elderly European Population
	Cubas De Basterrechea et al., 2020	Adherencia a la guía de alimentación saludable de la Sociedad Española de Nutrición Comunitaria (SENC) (2018) en personas mayores no institucionalizadas de Santander, España
	Machón et al., 2021	Proximity to facilities and its association with the health-related habits of functionally independent older adults
	Madeira et al., 2020	The association between dietary patterns and nutritional status in community-dwelling older adults—the PEN-3S study
	Neel Ocean et al. 2019	Lettuce be happy: A longitudinal UK study on the relationship between fruit and vegetable consumption and well-being
	Sanchez et al., 2019	Multiple factor analysis of eating patterns to detect groups at risk of malnutrition among home-dwelling older subjects in 2015
	Ferrand et al., 2018	Dietary patterns in french home-living older adults: Results from the PRAUSE study
	Whitelock et al., 2018	On your own: older adults' food choice and dietary habits
	Piumatti et al., 2018	Self-rated health among older adults in two fast ageing European countries: evidence from Italy and Serbia
	Bloom et al., 2017	Influences on diet quality in older age: the importance of social factors ILSE
	Jentsch et al., 2017	Typical patterns of modifiable health risk factors (MHRFs) in elderly

	women in Germany: Results from the cross-sectional German Health Update (GEDA) study, 2009 and 2010
Mendoca et al., 2016	Macronutrient intake and food sources in the very old: analysis of the Newcastle 85+ Study
Andreeva et al., 2016	Sex-specific sociodemographic correlates of dietary patterns in a large sample of French elderly individuals
Martín et al., 2015	Adherencia a la dieta mediterránea y su relación con el estado nutricional en personas mayores
Galiot e Cambrodón., 2015	Calidad de la dieta de la población española mayor de 80 años no institucionalizada
Granic A et al., 2015	Dietary patterns and socioeconomic status in the very old: The newcastle 85+ study
Selivanova et al., 2014	The relationship between healthy behaviors and health outcomes among older adults in Russia.
Eysteinsdottir et al., 2012	Assessing validity of a short food frequency questionnaire on present dietary intake of elderly Icelanders
Marisca-arcas et al., 2011	Validation of questionnaires to estimate adherence to the mediterranean diet and life habits in older indiViduals in southern spain
Thompson et al., 2010	Food shopping habits, physical activity and health-related indicators among adults aged ≥70 years
Cuervo et al., 2008	Food Consumption Analysis in Spanish Elderly Based upon the Mini Nutritional Assessment Test.
Pala et al., 2006	Associations between dietary pattern and lifestyle, anthropometry and other health indicators in the elderly participants of the EPIC-Italy cohort
Grammatikopoulou et al., 2006	Dietary Intake of Free-Living Elderly in Northern Greece
Eiben et al., 2003	Secular trends in diet among elderly Swedes – cohort comparisons over three decades
Gustafsson et al., 2002	Food-related health perceptions and food habits among older women.
Pryer et al., 2000	Identification of groups who report similar patterns of diet among a representative national sample of British adults aged 65 years of age or more
Bates CJ et al., 1999	Gender differences in food and nutrient intakes and status indices from the National Diet and Nutrition Survey of PeopleAged 65 Years and Over
Schroll et al., 1996	Food patterns of elderly Europeans
Whichelow et al., 1996	Dietary patterns and their associations with demographic, lifestyle and health variables in a random sample of British adults
Rothenberg et al., 1996	Food habits and nutrient intake in three 70-year-old free-living

		populations in Gothenburg, Sweden. A 22-year cohort study
	Trichopoulou et al, 1995	Diet and survival of elderly Greeks: A link to the past
	Lisette et al., 1992	Nutrition and Health of Elderly People in Europe: The EURONUT-SENECA Study
	Fernández-Ballart et al., 1989	Consumo, hábitos alimentarios y estado nutricional de la población de Reus. La dieta y el equilibrio nutricional en los mayores de 60 años
	Nissinen et al., 1982	Health behaviour of an elderly population in eastern Finland in 1982.
Wrong exposure (No data about determinants)	Waijers et al, 2021	Dietary patterns and survival in older Dutch women
	Samuelsson et al, 2019	Time trends in nutrient intake and dietary patterns among five birth cohorts of 70- year-olds examined 1971–2016: results from the Gothenburg H70 birth cohort studies, Sweden
	SantoS et al., 2014	Diet quality in elderlyportuguese households
	Wyka et al., 2010	nutritional determination of the health status in polish elderly people from an urban environment
	Robinson et al., 2009	Current patterns of diet in community-dwelling older men and women: results from the Hertfordshire Cohort Study
	Alstad T et al., 2006	Patterns of carbohydrate intake – a study of typology, associations and changes over time in an elderly swedish population
	Rurik y M Antal., 2003	Nutritional habits and lifestyle practice of elderly people in Hungary
	Hurson M et al., 1997	Dietary intakes in Ireland of a healthy elderly population
Don't met population criteria	Pagliai et al., 2018	Mediterranean diet, food consumption and risk of late-life depression: the mugello study.
	Conklin et al., 2015	Gender and the double burden of economic and social disadvantages on healthy eating: cross-sectional study of older adults in the EPIC-Norfolk cohort
	A Conklin et al 2014	Variety more than quantity of fruit and vegetable intake varies by socioeconomic status and financial hardship. Findings from older adults in the EPIC cohort
	Knudsen et al., 2014	Identifying dietary patterns and associated health-related lifestyle factors in the adult Danish population
	Wylie et al., 1999	Health and social factors affecting the food choice and nutritional intake of elderly people with restricted mobility
	Wichelow., 1996	Dietary patterns and their associations with demographic, lifestyle and health variables in a random sample of British adults
Other language	Araújo et al., 2015	Estilos de vida e percepçao do estado de saúde em idosos portugueses de zonas rural e urbana.

Table S3. Completary list of articles included for the NHLBI quality assessment

Author	Title	Score
L Serra Majem et al, 2000	[Food consumption and food sources of energy and nutrients in Canary Islands (1997-98)] - PubMed	7
A Øvrum et al, 2014	Age and socioeconomic inequalities in health: Examining the role of lifestyle choices	7
E Tourlouki et al, 2014	Are Current Dietary Habits in Mediterranean Islands a Reflection of the Past? Results from the MEDIS Study	6
Joost Oude Groeniger et al, 2019	Are socio-economic inequalities in diet and physical activity a matter of social distinction? A cross-sectional study	7
KM Appleton et al, 2010	Barriers to increasing fruit and vegetable intakes in the older population of Northern Ireland: low levels of liking and low awareness of current recommendations<br>Katherine	7
BA Holmes et al, 2011	Diet quality and the influence of social and physical factors on food consumption and nutrient intake in materially deprived older people	7
Corrêa Leite M et al, 2003	Dietary and nutritional patterns in an elderly rural population in Northern and Southern Italy: (I). A cluster analysis of food consumption ML	7
R. M. Ortega et al, 1995	Dietary assessment of a group of elderly Spanish people	5
D.M. SantoS et al, 2015	Dietary availability in elderly Portuguese households<br>Débora	5
P Huijbregts et al, 1995	Dietary intake in five ageing cohorts of men in Finland, Italy and the Netherlands	7
L Rasanen et al, 1992	Dietary intake of 70- to 89-year-old men in eastern and western Finland	6
P Amiano et al, 1999	Dietary intake of vegetables and fruits among adults in regions of Spain	7
C Bamia et al, 2005	Dietary patterns among older Europeans: the EPIC-Elderly study	7
D.M. SantoS et al, 2014	EFFECT OF SOCIODEMOGRAPHIC VARIABLES AND TIME ON FOOD GROUP CONTRIBUTION TO TOTAL FOOD AVAILABILITY IN PORTUGUESE ELDERLY HOUSEHOLDS	6
Robert Gajda et al, 2020	Elderly Perception of Distance to the Grocery Store as a Reason for Feeling Food Insecurity—Can Food Policy Limit This?	7
E Rothenberg et al, 1994	Food habits, food beliefs and socio-economic factors in an elderly population	7
ANGELA J et al, 1998	Gender and Living Alone as Determinants of Fruit and Vegetable Consumption among the Elderly Living at Home in Urban Nottingham	7
E Mertens et al, 2019	Geographic and socioeconomic diversity of food and nutrient intakes: a comparison of four European countries	7
K MORGAN et al, 2000	Healthy ageing in urban and rural Britain: a comparison of exercise and diet	7
S. Hawkesworth et al, 2017	Investigating the importance of the local food environment for fruit and vegetable intake in older men and women in 20 UK towns: a cross-sectional analysis of two national cohorts using novel methods	7
Imre Rurik et al, 2006	Nutritional Differences between Elderly Men and Women	6

F Contaldo et al, 1987	Nutritional status of the elderly in Naples	6
L. Mc Morrow et al, 2016	Perceived barriers towards healthy eating and their association with fruit and vegetable consumption	7
M Celodina et al, 2020	Retirement and Healthy Eating*	6
A Baker et al, 2003	Sex differences in fruit and vegetable intake in older adults	7
X Irz et al, 2014	Sociodemographic determinants of diet quality of the EU elderly: a comparative analysis in four countries	6
M Behanova et al, 2015	The effect of neighbourhood unemployment on health-risk behaviours in elderly differs between Slovak and Dutch cities	7
A Saba y M Vassallo, 2012	The influence of health involvement and satisfaction on healthy food choices among people over 60 years	6
S Maisey et al, 1995	Variation in food group and nutrient intake with day of the week in an elderly population	7
Sharon Friel et al, 2004	Who eats four or more servings of fruit and vegetables per day? Multivariate classification tree analysis of data from the 1998 Survey of Lifestyle, Attitudes and Nutrition in the Republic of Ireland	7

Table S4. Other factors associated to fruit and vegetables consumption

	Psychological status	Seasonality	Cooking skills	Smoking	Chew
E. van der Toorn et al., 2020		No association			
Boehm et al., 2018	High well-being was associated with more fruit and vegetable consumption RR 1.09 (1.07-1.12)				
Oliveira et al., 2014	No association				
Perna et al., 2012	Resilience was positively associated with high fruit and vegetable consumption				
Holmes et al., 2008			Cooking skills were associated with higher consumption of vegetables	Current smokers consumed significantly less fruit	Ability to chew was associated with highest fruit and vegetable consumption
Jonhson et al., 1998				Smoking was associated with less fruit	

Section and Topic	Item #	Checklist item	Location where item is reported
**TITLE**			
Title	1	Identify the report as a systematic review.	Title
**ABSTRACT**			
Abstract	2	See the PRISMA 2020 for Abstracts checklist.	Abstract (see below)
**INTRODUCTION**			
Rationale	3	Describe the rationale for the review in the context of existing knowledge.	Introduction, paragraphs 1, 2 and 3
Objectives	4	Provide an explicit statement of the objective(s) or question(s) the review addresses.	Introduction, paragraph 4
**METHODS**			
Eligibility criteria	5	Specify the inclusion and exclusion criteria for the review and how studies were grouped for the syntheses.	Methods, paragraph 2 and 3.
Information sources	6	Specify all databases, registers, websites, organizations, reference lists and other sources searched or consulted to identify studies. Specify the date when each source was last searched or consulted.	Methods, paragraph 2
Search strategy	7	Present the full search strategies for all databases, registers and websites, including any filters and limits used.	Methods, paragraph 3 and Table S1
Selection process	8	Specify the methods used to decide whether a study met the inclusion criteria of the review, including how many reviewers screened each record and each report retrieved, whether they worked independently, and if applicable, details of automation tools used in the process.	Methods, paragraph 2 and Figure 1.
Data collection process	9	Specify the methods used to collect data from reports, including how many reviewers collected data from each report, whether they worked independently, any processes for obtaining or confirming data from study investigators, and if applicable, details of automation tools used in the process.	Methods, paragraph 5
Data items	10a	List and define all outcomes for which data were sought. Specify whether all results that were compatible with each outcome domain in each study were sought (e.g. for all measures, time points, analyses), and if not, the methods used to decide which results to collect.	Methods, paragraph 5
	10b	List and define all other variables for which data were sought (e.g. participant and intervention characteristics, funding sources). Describe any assumptions made about any missing or unclear information.	Methods, paragraph 5
Study risk of bias assessment	11	Specify the methods used to assess risk of bias in the included studies, including details of the tool(s) used, how many reviewers assessed each study and whether they worked independently, and if applicable, details of automation tools used in the process.	Methods, paragraph 4
Effect measures	12	Specify for each outcome the effect measure(s) (e.g. risk ratio, mean difference) used in the synthesis or presentation of results.	Not applicable

Synthesis methods	13a	Describe the processes used to decide which studies were eligible for each synthesis (e.g. tabulating the study intervention characteristics and comparing against the planned groups for each synthesis (item #5)).	Methods, Paragraph 5
	13b	Describe any methods required to prepare the data for presentation or synthesis, such as handling of missing summary statistics, or data conversions.	Not applicable

Section and Topic	Item #	Checklist item	Location where item is reported
	13c	Describe any methods used to tabulate or visually display results of individual studies and syntheses.	Methods, paragraph 5
	13d	Describe any methods used to synthesize results and provide a rationale for the choice(s). If meta-analysis was performed, describe the model(s), method(s) to identify the presence and extent of statistical heterogeneity, and software package(s) used.	Not applicable
	13e	Describe any methods used to explore possible causes of heterogeneity among study results (e.g. subgroup analysis, meta-regression).	Not applicable
	13f	Describe any sensitivity analyses conducted to assess robustness of the synthesized results.	Not applicable
Reporting bias assessment	14	Describe any methods used to assess risk of bias due to missing results in a synthesis (arising from reporting biases).	Not reported
Certainty assessment	15	Describe any methods used to assess certainty (or confidence) in the body of evidence for an outcome.	Not reported
**RESULTS**			
Study selection	16a	Describe the results of the search and selection process, from the number of records identified in the search to the number of studies included in the review, ideally using a flow diagram.	Results, paragraph 1 and figure 1
	16b	Cite studies that might appear to meet the inclusion criteria, but which were excluded, and explain why they were excluded.	Table S2
Study characteristics	17	Cite each included study and present its characteristics.	Results, table 2 and table 3
Risk of bias in studies	18	Present assessments of risk of bias for each included study.	Results, paragraph 2, Figure 2 and Table 1
Results of individual studies	19	For all outcomes, present, for each study: (a) summary statistics for each group (where appropriate) and (b) an effect estimates and its precision (e.g. confidence/credible interval), ideally using structured tables or plots.	Results table 4-5
Results of syntheses	20a	For each synthesis, briefly summarize the characteristics and risk of bias among contributing studies.	Results, paragraph 2 and 3
	20b	Present results of all statistical syntheses conducted. If meta-analysis was done, present for each the summary estimate and its precision (e.g. confidence/credible interval) and measures of statistical heterogeneity. If comparing groups, describe the direction of the effect.	Not applicable
	20c	Present results of all investigations of possible causes of heterogeneity among study results.	Not applicable

Section and Topic	Item #	Checklist item	Location where item is reported
	20d	Present results of all sensitivity analyses conducted to assess the robustness of the synthesized results.	Not applicable
Reporting biases	21	Present assessments of risk of bias due to missing results (arising from reporting biases) for each synthesis assessed.	Not applicable
Certainty of evidence	22	Present assessments of certainty (or confidence) in the body of evidence for each outcome assessed.	Results, paragraph 4-5
**DISCUSSION**			
Discussion	23a	Provide a general interpretation of the results in the context of other evidence.	Paragraph 1–4
	23b	Discuss any limitations of the evidence included in the review.	Paragraph 5
	23c	Discuss any limitations of the review processes used.	Paragraph 5
	23d	Discuss implications of the results for practice, policy, and future research.	Paragraph 6
**OTHER INFORMATION**			
Registration and protocol	24a	Provide registration information for the review, including register name and registration number, or state that the review was not registered.	No
	24b	Indicate where the review protocol can be accessed, or state that a protocol was not prepared.	No
	24c	Describe and explain any amendments to information provided at registration or in the protocol.	No
Support	25	Describe sources of financial or non-financial support for the review, and the role of the funders or sponsors in the review.	Funding
Competing interests	26	Declare any competing interests of review authors.	Conflicts of interest
Availability of data, code and other materials	27	Report which of the following are publicly available and where they can be found: template data collection forms; data extracted from included studies; data used for all analyses; analytic code; any other materials used in the review.	All information are included in the Manuscript and suppliment ary material

PRISMA 2020 for Abstracts Checklist

Section and Topic	Item #	Checklist item	Reported (Yes/No)
**TITLE**			
Title	1	Identify the report as a systematic review.	Yes
**BACKGROUND**			
Objectives	2	Provide an explicit statement of the main objective(s) or question(s) the review addresses.	Yes
**METHODS**			
Eligibility criteria	3	Specify the inclusion and exclusion criteria for the review.	Yes
Information sources	4	Specify the information sources (e.g. databases, registers) used to identify studies and the date when each was last searched.	Yes
Risk of bias	5	Specify the methods used to assess risk of bias in the included studies.	Yes
Synthesis of results	6	Specify the methods used to present and synthesize results.	Yes
**RESULTS**			
Included studies	7	Give the total number of included studies and participants and summarise relevant characteristics of studies.	Yes
Synthesis of results	8	Present results for main outcomes, preferably indicating the number of included studies and participants for each. If meta-analysis was done, report the summary estimate and confidence/credible interval. If comparing groups, indicate the direction of the effect (i.e. which group is favoured).	Yes
**DISCUSSION**			
Limitations of evidence	9	Provide a brief summary of the limitations of the evidence included in the review (e.g. study risk of bias, inconsistency and imprecision).	No
Interpretation	10	Provide a general interpretation of the results and important implications.	Yes
**OTHER**			
Funding	11	Specify the primary source of funding for the review.	No
Registration	12	Provide the register name and registration number.	NA
